# Combating resilient *Acanthamoeba castellanii* cysts: Ultrasonic frequency-dependent effects on viability and excystment

**DOI:** 10.1016/j.ultsonch.2025.107695

**Published:** 2025-11-28

**Authors:** Prince Nana Amaniampong, Ascel Samba-Louaka

**Affiliations:** a CNRS, Université de Poitiers, Institut de Chimie des Milieux et Matériaux de Poitiers (IC2MP) (ENSI-Poitiers), B1, 1 rue Marcel Doré, 86073 Poitiers, France; b Université de Poitiers, Laboratoire Ecologie et Biologie des Interactions, UMR Centre National de la Recherche Scientifique 7267, Bât B31, 3 rue Jacques Fort, 86073 Poitiers, France

**Keywords:** Amoeba, Ultrasound, High frequency, Low frequency

## Abstract

Free-living amoebae (FLA) are significant environmental reservoirs for numerous pathogenic microorganisms. These protists exhibit remarkable resilience, often evading biocides due to their ability to form highly resistant dormant cysts under unfavorable conditions. This study investigated the effects of ultrasound treatment on the viability of these amoeba cysts, using *Acanthamoeba castellanii*, a prevalent water and soil amoeba, as a model. We found that low-frequency ultrasound treatment significantly impaired the revival of *Acanthamoeba* cysts. In contrast, high-frequency ultrasound effectively ruptured cyst walls and induced direct amoebal death. Further investigation using *tert*-Butanol, an OH radical scavenger, partially mitigated the effects induced by high-frequency ultrasound, demonstrating that reactive oxygen species contribute to amoeba damages. Our results highlight ultrasound’s significant potential as an efficient and effective strategy to combat resilient *Acanthamoeba* cysts.

## Highlights

• Low frequency ultrasound can damage *A. castellanii* cysts and impair *A. castellanii* excystment.
• High frequency ultrasound induces destruction of *A. castellanii* cysts.
• Reactive oxygen species contributes to cysts breaks provoked by ultrasound.

## Graphical abstract

**Figure:**
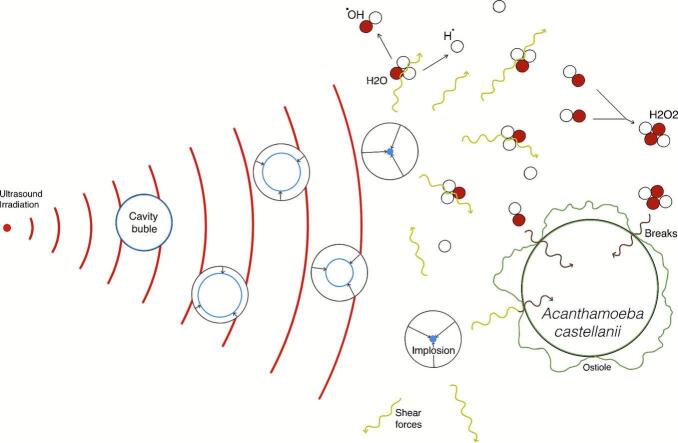

## 1 Introduction

Free-living amoebae (FLA) are unicellular organisms commonly found in diverse environment [1]. They prey on and ingest microorganisms, including bacteria, fungi, and viruses, through phagocytosis, thereby structuring microbial communities. Certain microorganisms can resist and escape amoebal digestion; these amoeba-resistant bacteria (ARB) have been shown to become more virulent to macrophages and more resistant to biocides after escaping amoebae [2]. Thus, FLA serve as “training grounds” and reservoirs for potentially pathogenic bacteria [[2], [3], [4]]. Furthermore, some FLA are themselves direct causative agents of human diseases; for example, *Acanthamoeba* species cause granulomatous encephalitis and vision-threatening keratitis [5]. Their removal from anthropogenic environments is therefore a significant public health concern. Under unfavorable conditions such as desiccation, starvation, and osmotic changes, amoebae trophozoites differentiate into resting, highly resistant forms known as cysts, enabling their survival in hostile environments. This process is reversible, with amoebae excysting under favorable conditions [6]. Amoebal cysts are enveloped by a robust wall composed of proteins and carbohydrates, conferring increased resistance to physical and chemical treatments. Furthermore, microorganisms internalized within these cysts may be protected from disinfection treatments, allowing their persistence in the environment [7].

Currently, FLA colonizing anthropogenic environments are targeted by various disinfection protocols [8]*.* Nevertheless, reports highlight varied FLA resistance to disinfection treatments. For example, while *Acanthamoeba* species cysts are sensitive to moist heat at 65 °C [9] they can resist exposure to chlorine, UV, X-ray, and gamma irradiation [8]. This selective resistance to established disinfectants underscores the urgent need for novel amoebicidal treatments. Ultrasound has demonstrated efficacy in inactivating various microorganisms, including bacteria and fungi [10]. Its biocidal effects stem from both mechanical forces and sonochemical reactions generated by acoustic cavitation [11]. The collapse of cavitation bubbles produces shear forces and energy that damage cells [12]. This energy also ruptures chemical bonds and, in aqueous environments, generates reactive oxygen species like hydroxyl radicals (^•^OH) and hydrogen peroxide (H_2_O_2_) that attack cellular components, leading to inactivation [13]. Interestingly, reactive oxygen species have also been reported to damage the resistant cyst wall structure of the protozoan Giardia intestinalis, thereby reducing its viability [14]*.*

To our knowledge, very few studies have reported on the ultrasonic irradiation of amoebae. Holmer et al. reported cell membrane damage in *Amoeba proteus* exposed to 1 MHz ultrasound [15]. However, no difference in growth was found for *Acanthamoeba castellanii* trophozoites in gelled suspensions exposed to 1 MHz ultrasound at 10 °C compared to control cells [16]. In contrast, Priscilla Declerck et al. observed destruction of *A. castellanii* trophozoites at 36 kHz but no effects on cyst viability [17]. Given the scarcity and varied findings regarding ultrasound effects on amoebae, our study aimed to comprehensively explore the effects of both low and high-frequency ultrasound on *A. castellanii* cysts. This work demonstrates that cavitation bubbles act as potent micro-weapons, effectively destroying *A. castellanii* cysts under near-ambient conditions.

## 2 Materials and methodology

### 2.1 *A. castellanii* culture and cyst formation

*Acanthamoeba castellanii* trophozoites (ATCC 30010) were cultured at room temperature in cell culture flasks containing PYG medium (2 % proteose peptone, 0.1 % yeast extract, 0.1 M glucose, 0.1 % sodium citrate dihydrate, 0.4 mM CaCl_2_, 4 mM MgSO_4_, 0.05 mM Fe(NH_4_)_2_(SO_4_)_2_ 6H_2_O, 2.5 mM K_2_HPO_3_, 2.5 mM NaH_2_PO_3_, pH 6.5). Upon reaching confluency, cells were washed and then incubated for one month in an encysting medium (0.1 M KCl, 0.4 mM CaCl_2_, 1 mM NaHCO_3_, 8 mM MgSO_4_, and 20 mM 2-amino-2-methyl-1,3-propanediol, pH 8.8). The resulting cysts were recovered, suspended in sterile distilled and deionized water for 16 h, and adjusted to a final concentration of approximately 30 × 10^4^ cysts/mL in a 50 mL volume.

For excystment tests, cysts were incubated in PYG medium at 30 °C.

### 2.2 Ultrasound treatments

For low-frequency ultrasound treatment, around 30 × 10^4^ *A. castellanii* cysts per millilitre, in a 50 mL of deionized water, were exposed to 20 kHz at 20 % amplitude using a Branson Digital Sonifier 450. For high-frequency treatment, cells underwent ultrasonic irradiation at 500 kHz with a NexTgen LAB1000 (Sinaptec) system in a final volume of 50 mL of deionized water. Similarly, to the low-frequency ultrasound treatment, the initial concentration was approximately 30 × 10^4^ *A. castellanii* cysts per millilitre. The calorimetrically determined acoustic power density was 0.26 W/mL for high-frequency ultrasound and 0.21 W/mL for low-frequency ultrasound. The cell suspension temperature was maintained at 22 °C using an external chiller. To determine cell concentration, *A. castellanii* were harvested and counted for each experimental condition using FastRead 102 plastic counting slides (Biosigma).

### 2.3 Videomicroscopy

#### 2.3.1 Imaging for low-frequency ultrasound treatment

After low-frequency ultrasound treatment, *A. castellanii* cysts were seeded into a µ-slide 8-well plate (ibidi®) at a density of 2.5 × 10^4^ cells per well. These were cultured in PYG medium at room temperature. Images were captured using an Andor Revolution XD imaging system, featuring a CSUX1 Yokogawa spinning disk unit and an Andor Ixon + 897 back-illuminated EMCCD camera (16 µm^2^ pixel size). This system was mounted on an Olympus inverted IX81S1F-ZDC microscope equipped with a 40x objective lens (NA 0.95). IQ3 acquisition software (Andor Technology) was used to acquire approximately 1,800 images, each 512x512 pixels, over a 16-hr period.

#### 2.3.2 Imaging for high-frequency ultrasound treatment

For cells exposed to high-frequency ultrasound, 50 µL of the post-treatment cell suspension was added to a µ-slide 8-well plate (ibidi®) and supplemented with 250 µL of PYG medium. Cells were then incubated at room temperature for 48 h. Images were subsequently acquired every three minutes for 16 h using a Nikon CrestOptics X-Light V3 Spinning Disk Confocal microscope with a 40× objective lens. Nikon Imaging Software was used to capture 2720 × 2720 pixel images.

## 3 Results

### 3.1 Low frequency ultrasound impairs *A. castellanii* excystment

**Fig. 1:**
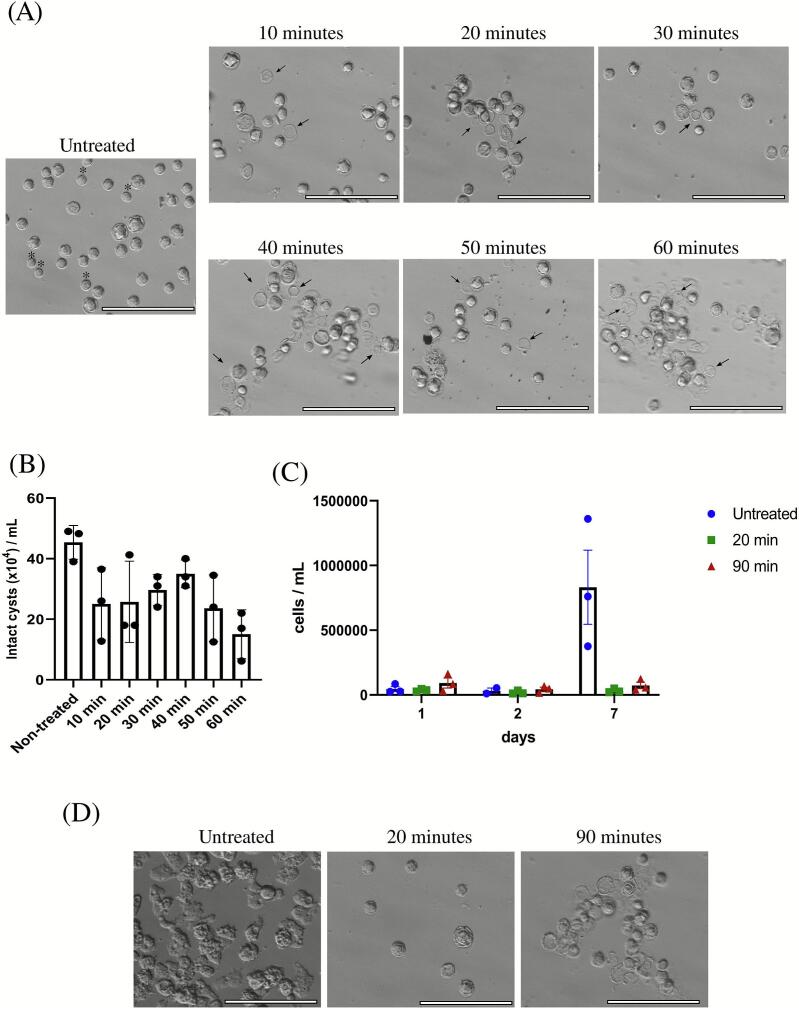
**Low frequency ultrasound damage *A. castellanii* cysts and delay excystment.** (A) *A. castellanii* cysts were exposed to low frequency ultrasound and cells were harvested for imaging at the indicated time-points. * Corresponds to pseudocysts or immature cysts and damaged cysts are indicated with arrows. (B) Concentration of *A. castellanii* cyst was estimated and results come from 3 independent experiments (mean +/− SEM, p = 0.0938). (C) 12.10^4^ cysts exposed to low frequency ultrasound for 20 or 90 min were incubated within the growing medium. Cells were harvested for counting at the indicated time-points. Regarding the last time-point (7 days after the incubation in the growing medium), cells were also imaged (panel D). Data comes from 3 independent experiments (mean +/− SEM, p = 0.9929). For all the statistical analyses, we used the Kruskal-Wallis test and scale bars correspond to 100 µm.

To evaluate the effects of low-frequency ultrasound on *A. castellanii* cysts, cells underwent treatment for 1.5 h. Every 20 min, a 2 mL aliquot of the cell suspension was collected for counting and imaging. Initially, the suspension contained a mix of mature cysts (characterized by a double-layered membrane) and some pseudo or immature cysts, which displayed only a single membrane (Fig. 1A). Remarkably, after just 20 min of low-frequency ultrasound treatment, we observed the presence of empty shells and damaged cells (Fig. 1A). From 60 to 90 min, significant aggregation of cells was noted, likely resulting from medium evaporation in the sonochemical experiments.

We quantified undamaged cysts and found that low-frequency ultrasound reduced the concentration of intact *A. castellanii* cysts (Fig. 1B). However, this reduction was not significantly different compared to untreated cells. We assessed the ability of *A. castellanii* cysts to excyst after exposure to low-frequency ultrasound. Seven days after incubation in a growth medium, we observed a significant increase in cell number for untreated cysts (Fig. 1C), indicating successful excystment and differentiation into trophozoites (Fig. 1D). In stark contrast, cysts exposed to low-frequency ultrasound for either 20 or 90 mins remained almost entirely rounded. Furthermore, a videomicroscopy experiment, initiated immediately after adding the growth medium, showed the emergence of trophozoites in untreated conditions after approximately 16 h (see supplementary video: “untreated cells”). Conversely, very few trophozoites appeared in cultures treated with low-frequency ultrasound for 90 mins (see supplementary videos: “20 min low frequency ultrasound” and “90 min low frequency ultrasound”). These findings collectively suggest that low-frequency ultrasound delays the excystment of *A. castellanii* cysts.

### 3.2 High frequency ultrasound break *A. castellanii* cysts via reactive oxygen species

**Fig. 2:**
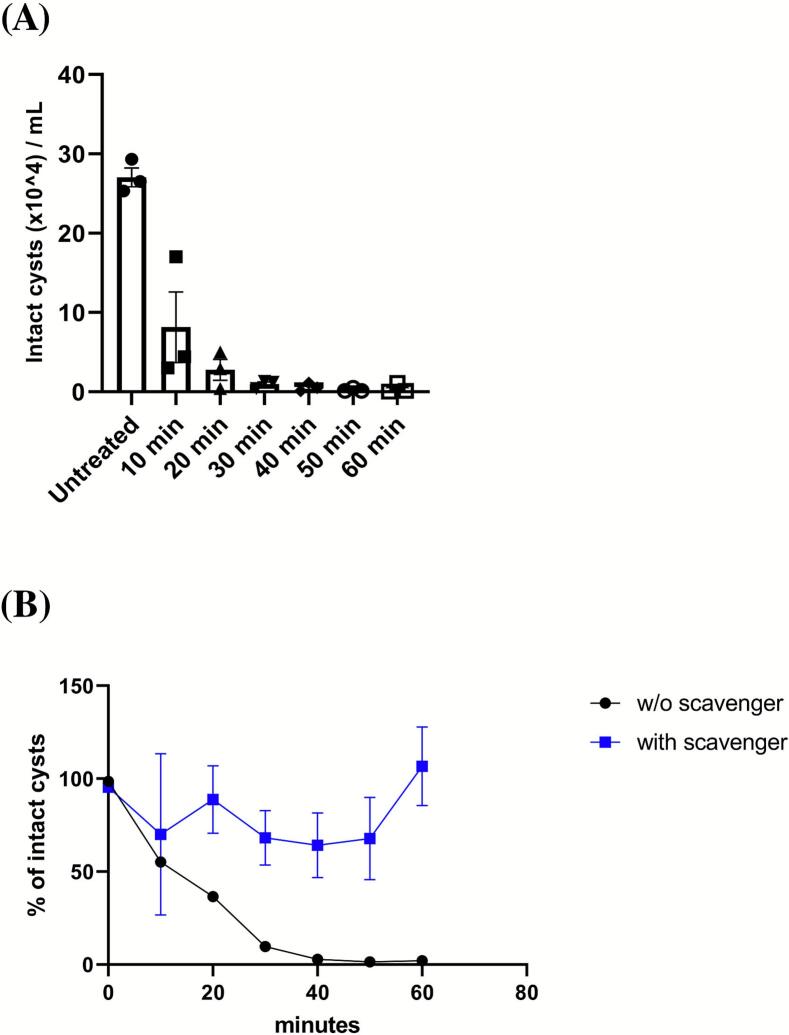
**High frequency ultrasound destroys *A. castellanii* cysts.** (A) *A. castellanii* cysts were exposed to high frequency ultrasound and some cells were harvested for counting at different time-points. (B) *A. castellanii* cysts were treated with high frequency ultrasound in presence of 1 % of *tert*-butanol. Results come from 3 independent experiments (mean +/- SEM). For the statistical analysis, (A) we used the Kruskal-Wallis, p = 0.0183 and (B) we used a Mann Whitney test, p = 0.0175, to compare samples with and without scavenger.

Next, we evaluated the effects of high-frequency ultrasound on *A. castellanii* cysts over one hour, collecting 2 mL aliquots of the cell suspension every ten minutes for counting and imaging. We observed that high-frequency ultrasound significantly destroyed *A. castellanii* cysts (Fig. 2A). Given that high-frequency ultrasound generates more free radicals than its low-frequency counterpart [[18], [19], [20]], we treated *A. castellanii* cysts in the presence of *tert*-butanol, a hydroxyl radical scavenger. As shown in Fig. 2B, the presence of *tert*-butanol significantly impaired cyst destruction compared to conditions without the scavenger. We hypothesize that the slight increase in intact cysts observed at 60 min with the scavenger resulted from minor medium evaporation during the experiment. *Tert*-butanol appeared not to affect amoeba physiology, as some cysts could still excyst into motile trophozoites after 48 h in a growth medium (see supplementary videos: “20 min high frequency ultrasound” and “20 min high frequency ultrasound + scavenger”). These results strongly suggest that reactive oxygen species contribute to amoeba damages induced by high-frequency ultrasound.

## 4 Discussion

This work aimed to assess the effects of both low and high-frequency ultrasound on *Acanthamoeba castellanii* cysts. Although cysts from *G. intestinalis* have been shown to be inactivated by mid- to high-frequency ultrasound, we explored how another protozoan with a different cyst wall structure might respond to ultrasound. The *Acanthamoeba* cyst wall is more complex than the single-layered cyst wall of *Giardia*, which is composed of β-1,3-linked N-acetylgalactosamine (GalNAc) with some lectins. By contrast, the *Acanthamoeba* cyst wall forms a double-walled envelope consisting of two microfibril-dense layers (the outer ectocyst and the inner endocyst), which are rich in cellulose and lectins [21]. Our data clearly demonstrate that high-frequency ultrasound effectively damages *A. castellanii* cysts within just 10 min. This potent effect is, in part, mediated by reactive oxygen species, as evidenced by the partial protection offered when *tert*-butanol, an OH radical scavenger, was present. In contrast, while some amoebal damage was observed with low-frequency ultrasound, we didn't quantify a significant cyst reduction compared to untreated cells. This finding aligns with a previous publication reporting no significant effects of ultrasound on overall cyst viability [16]. The absence of a strong cysticidal effect by low-frequency ultrasound could stem from the robust nature of the cyst wall, as low-frequency ultrasound efficacy has been shown to depend on the presence or absence of resistant microbial structures like capsules [10]. Therefore, we hypothesize that low-frequency ultrasound primarily affected only immature or pseudocysts, explaining the slight decrease in observed cyst numbers. Nevertheless, low-frequency ultrasound was notably able to impair *A. castellanii* excystment, suggesting it still imposes significant stress on the amoebae. Furthermore, we observed an aggregation of cysts, a phenomenon previously reported by Holmer et al. [14]. Further analysis of the amoeba cyst wall is necessary to identify any modifications induced by ultrasound.

### 4.1 Differential mechanisms of action

The observed difference in efficacy between the two ultrasound frequencies mirrors findings in bacterial destruction, where high-frequency ultrasound often proves more efficient due to enhanced radical oxygen species generation, specifically hydroxyl radical [10]. Hydroxyl radicals, known for their production under high-frequency ultrasound [22], are known to attack microbial cell membranes, leading to bactericidal action. While commercial products containing 3 % hydrogen peroxide were ineffective against *Acanthamoeba* spp. for 30 mins [18,19], they became cysticidal after 4 hr [20]. The rapid cyst destruction (within 10 min) achieved by high-frequency ultrasound in our study suggests a synergistic action between its mechanical and chemical effects induced by ultrasonic cavitation bubbles implosions. This need for such a synergy could explain the weaker cyst destruction observed with low-frequency ultrasound, as it generates fewer radicals compared to high-frequency applications.

## 5 Conclusion

This study aimed to determine the effects of low and high-frequency ultrasound treatment on *A. castellanii* cysts. Both frequencies demonstrated the ability to damage amoeba cysts, with high-frequency ultrasound exhibiting a significantly stronger effect, partly attributable to the generation of reactive oxygen species. These findings suggest that ultrasound represents a promising alternative technology for eliminating free-living amoebae.

## CRediT authorship contribution statement

**Prince Nana Amaniampong:** Writing – review & editing, Writing – original draft, Methodology, Investigation, Funding acquisition, Formal analysis. **Ascel Samba-Louaka:** Writing – review & editing, Writing – original draft, Visualization, Project administration, Investigation, Funding acquisition, Formal analysis, Conceptualization.

## Declaration of competing interest

The authors declare that they have no known competing financial interests or personal relationships that could have appeared to influence the work reported in this paper.
